# Time to under-five mortality and its predictors in rural Ethiopia: Cox-gamma shared frailty model

**DOI:** 10.1371/journal.pone.0266595

**Published:** 2022-04-06

**Authors:** Melaku Yalew, Mastewal Arefaynie, Gedamnesh Bitew, Erkihun Tadesse Amsalu, Bereket Kefale, Amare Muche, Zinabu Fentaw, Muluken Genetu Chanie, Mequannent Sharew Melaku, Bezawit Adane, Yitayish Damtie, Metadel Adane, Wolde Melese Ayele, Assefa Andargie, Reta Dewau

**Affiliations:** 1 Department of Reproductive and Family Health, School of Public health, College of Medicine and Health Sciences, Wollo University, Dessie, Ethiopia; 2 Department of Epidemiology and Biostatistics, School of Public health, College of Medicine and Health Sciences, Wollo University, Dessie, Ethiopia; 3 Department of Health Systems and Policy, School of Public health, College of Medicine and Health Sciences, Wollo University, Dessie, Ethiopia; 4 Department of Health Informatics, Institute of Public health, College of Medicine and Health Sciences, University of Gondar, Gondar, Ethiopia; 5 Department of Environmental Health, College of Medicine and Health Sciences, Wollo University, Dessie, Ethiopia; University of Botswana, BOTSWANA

## Abstract

**Background:**

Under-five mortality (U5M) is one of the most important and sensitive indicators of the health status of the community. Despite there having been a substantial reduction in U5M since 1990, its rate is still high in Sub-Saharan African countries. Thus, this study aimed to assess time to under-five mortality and its predictors in rural Ethiopia.

**Methods:**

This study utilized a secondary analysis of the 2016 Ethiopia Demographic and Health Survey (EDHS). A total of 9,807 weighted under-five children selected at different stages were included in the analysis. The Kaplan-Meier and Cox’s-gamma shared frailty models were used to estimate survival time and to identify predictors of under-five mortality, respectively. An adjusted Hazard Ratio (AHR) along with a 95% Confidence Interval (CI) was used to measure the effect size and direction of the association.

**Results:**

The study indicated that 6.69% (95% CI: 6.13, 7.30) of children died before celebrating their fifth birthday in rural Ethiopia. Of all the deaths, the median time to death was 27 months. After controlling the effect of cluster and other confounding factors, female sex (AHR = 0.62, 95% CI: 0.52, 0.75), ever born greater than five children (AHR = 1.40, 95% CI: 1.07, 1.83), very large size at birth (AHR = 1.33, 95% CI: 1.03 1.71), very small size at birth (AHR = 1.41, 95% CI: 1.10, 1.82), twin pregnancy (AHR = 3.5, 95% CI: 2.47, 4.88), not ever breastfeeding (AHR = 11.29, 95% CI: 9.03, 14.12), unimproved latrine (AHR = 3.44, 95% CI: 1.91, 6.17), covered by health insurance (AHR = 0.29, 95% CI: 0.12, 0.70) were predictors of under-five mortality.

**Conclusions:**

Still under-five mortality was high in rural Ethiopia as compared to the global under-five mortality rate. In the final model, sex of a child, the total number of children ever born, children’s size at birth, type of pregnancy, breastfeeding, type of toilet, and being covered by health insurance were significant predictors of under-five mortality. Further emphasis should be given to twin and not breastfeeding children, as well as households’ better encouraging membership of community health insurance and utilization of improved latrines.

## Introduction

Under-five mortality (U5M) is one of the most important sensitive indicators of the health status of the community. The global estimate showed 5.6 million children died before reaching their fifth birthday each year [1, 2]. Even though there has been a substantial reduction in child mortality in the past three decades, the progress has not been effective in Sub-Saharan Africa and South Asia [3]. According to a systematic analysis for the Global Burden of Disease Study from 1970–2016, the global under-five mortality rate (U5M) was found to be 4.10%, which needs major public health interventions [4]. Still, many developing countries are suffering from a high number of U5M [5]. Mainly, Sub-Saharan African countries and Southern Asian countries share over 80% of under-five deaths [6]. Children in those countries were 10 times more likely to die before 5 years than children in developed countries [7]. Particularly in Ethiopia, the 2016 EDHS report showed that U5M was 6.7% [8].

Under-five, mortality was significantly associated with maternal mental distress [9]. In addition, the rate of hospitalization was very high among mothers who had lost their child compared with those who had not [10]. Under-five mortality was influenced by numerous factors: child factors (age, sex, and size at birth) [11–15], household factors (wealth, family size, water source, and latrine type) [16–19], and community-level factors (residence, community-level of education, and wealth) [20, 21].

At least 80% of the Ethiopian population were rural residents, and life expectancy and age were relatively short among rural residents [22, 23]. Keeping this in mind, Ethiopia aimed to reduce the U5MR to 25 or less per 1000 live births by 2030 [24]. This aim will not be achieved unless the risk factors are extensively investigated and intervened upon. Although different studies have been conducted in Ethiopia regarding under-five mortality, all of them targeted both urban and rural populations, despite significant variations being documented [12, 25]. Furthermore, the data were correlated, even though previous studies on this data had ignored it [12, 26, 27]. Though some of the studies tried to consider the nature of the data, under-five mortality is time to the event of interest, and the analysis didn’t consider the effect of time [28, 29]. Modeling such types of data is better to account for both hierarchical and censoring data [11, 14, 17, 25, 28, 30–32]. Therefore, this study aimed to assess time to under-five mortality and its predictors in rural Ethiopia using survival analysis, accounting for the correlated nature of the data.

## Methods

### Study setting, population and data source

A community-based cross-sectional survey was conducted in Ethiopia. The survey was conducted from January 18 to June 27, 2016, by the Central Statistical Agency (CSA) in coordination with the Federal Minister of Health (FMoH) and the Ethiopia Public Health Institute (EPHI). The 2016 EDHS data were accessed from the DHS program after contacting them through formal registration. The source and study population were all under-five children in rural Ethiopia and all under-five children selected clusters of rural Ethiopia, respectively. The data set was limited to rural under-five children whose ages at death for the deceased and current age of a child for the living were recorded.

### Sample size determination and sampling procedure

A total of 9,807 weighted under-five children from EDHS 2016 dataset were included from nine geographical regions and two administrative cities of Ethiopia. The 2016 EDHS sample was stratified and selected in two stages. In the first stage, stratification was conducted by region, and then each region was stratified as urban and rural, yielding 21 sampling strata. A total of 645 EAs (enumeration areas) (202 in urban areas and 443 in rural areas) were selected with probability proportional to EA size in each sampling stratum. A household listing operation was carried out in all of the selected EAs.

**Fig 1:**
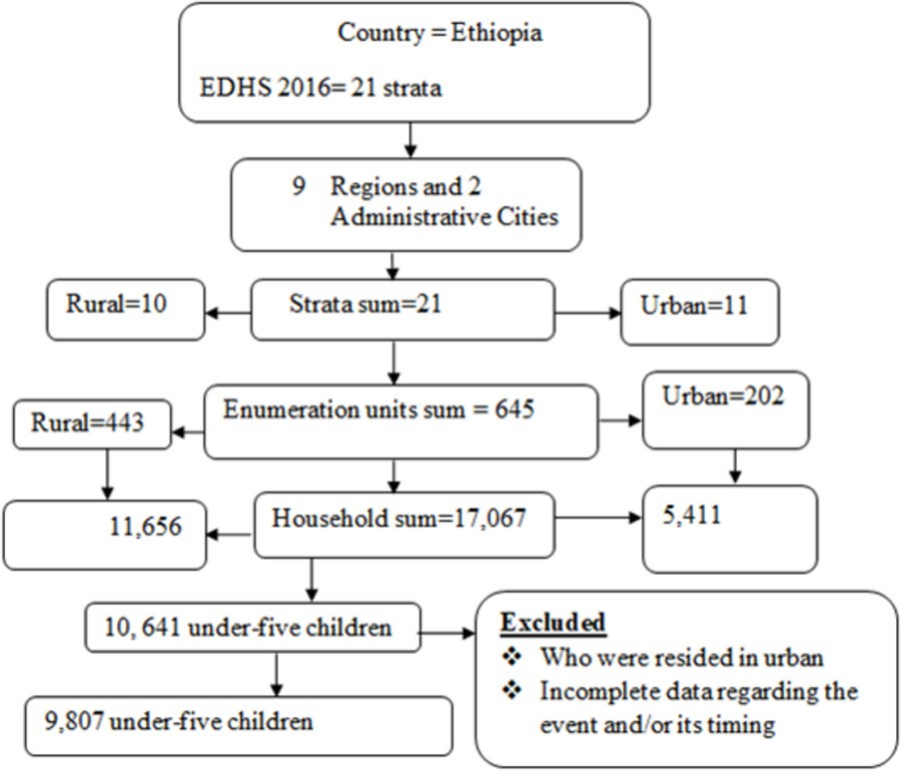
Sample size and schematic presentation of the sampling procedure for time to under-five mortality and its predictors in rural Ethiopia.

In the second stage, a fixed number of 28 households per cluster were selected with an equal probability systematic selection from the newly created household listing. Data coding and recoding was done to reach the exact number of under-five children in the 2016 EDHS. In addition, the timing of death for the deceased and age of the child for non-deceased was available with separate codes for all times with the respective type of respondent. Finally, by combing the above scenarios using the appropriate STATA command new variable was generated containing the total sample size which was 9,807 under-five children in the 2016 EDHS (Fig 1).

### Variable measurement

The dependent variable (time to death) was the age of a child in months when he/she died after live birth (beginning time). Similarly, events (uncensored) refer to children who died either before or at the time of the EDHS data collection period. Whereas "censored" refers to an event that didn’t happen (children alive) until the last EDHS data collection date [8]. Community-level variables were computed by aggregating individual-level variables in each cluster.

### Data quality control and analysis

A pre-test was conducted and necessary modifications were performed before the actual data collection period. The data collection instrument was transformed into the local language of the participants [8]. An initial exploratory data analysis was conducted to check for outliers, missing, and consistency after sample weighting. Stata/SE version 14.0 was used to analyze the data. A Log-rank test was used to assess statistical differences between categorical predictor variables in the outcome of interest over time. The Kaplan-Meier method was also used to estimate the time to under-five mortality. The Likelihood ratio chi-square and Wald chi-square tests were used to assess the significant contribution of clusters (frailty) on time to child mortality and for the overall model significance tests respectively. The Cox-gamma shared frailty model was used to identify predictors of under-five mortality. Shared gamma frailty analysis was done since the data was cluster level grouping, which was made between-group heterogeneity measured as theta chi-square test and was significant (p<0.001). So, this between-group heterogeneity creates unexploded variations. The hazard or risk of death was modeled using a mixed-effect model as follows [33]:

λϵ(t,x,w)=λΟ(t)exp⦃v(t,x)+w⦄Or

λϵ(t,x,u)=λΟ(t)uexp⦃v(t,x),

Where: λ*O* is the baseline hazard, x is the vector of covariates, v takes account the non-linear or non-proportional effects, w is the random effect defined at the cluster level and u is equal to exp⦃w⦄ is called the shared frailty.

Gamma distribution for the frailty, z, whose density is

$$u=g\;(z)=\frac{z^{\frac{1}{\left(\theta -1\right)}}exp^{-\frac{z}{\theta }}}{\gamma (\frac{1}{\theta })\theta^{\frac{1}{\theta }}},\;\theta \geq 0$$

And the mean value of the frailty is 1, and the variance of the frailty is θ, which may be used as a measure of association. Large values of θ reflect greater heterogeneity between subgroups and a stronger association among members of a subgroup [34].

In the analysis, the first uni-variable cox-gamma regression model was fitted, and variables with a p-value less than 0.2 were selected for multivariable cox-regression. Then, the analysis was done in four steps: Model 0 (null model or empty model) without any explanatory variables, model1 (only individual-level variables); model 2 (only cluster or community-level variables) and model 3 (both individual and community-level variables). The measure of association (fixed effect) was estimated by using Adjusted Hazard Ratio (AHR) and random effect with θ along with their 95% Confidence Interval (CI). In the final model, the level of statistical significance was set at a P-value of less than 0.05. Model adequacy was checked by using Cox-Snell residuals, which is a Nelson Aalen cumulative hazard function against the Cox-Snell residuals plot; a linear pattern making a straight line through the origin indicates the best fit model [35]. Some variability in the right-hand tail around the 45° line is due to a smaller effective sample due to prior early-age deaths and censoring. Log-likelihood ratio deviance test was also applied.

#### Ethical consideration

A written approval letter was obtained to use the EDHS dataset from the DHS program for the current study. The IRB also approved the dataset for public use without personal, household, or sample community identification. So, the privacy of the participants was kept anonymously. Moreover, per DHS program discipline, this data was used for the current study only. However, the data underlying the study can be accessed after legal registration at www.measuredhs.com and writing a convincing letter to the project for the DHS program.

## Results

### Household and parental characteristics

Three hundred fifty-nine (3.67%) of the children were from teenage mothers. Similarly, more than two-thirds (70.95%) of mothers of under-five children were not formally educated. More than half (57.40%) of children’s mothers were not working at the time of the survey. Regarding household wealth, 2572 (26.23%) of the mothers lived in the poorest wealth quintile. One thousand two hundred sixteen (12.40%) of the households were headed by women. Only, 324 (3.31%) and 527 (5.38%) of the households were covered by health insurance (Log-rang test: 8.88, p-value 0.003) and used improved latrines (Log-rank test: 4.37, p-value = 0.04) respectively. Seven thousand two hundred eighty-four (74.27%) of mothers had 3 or above ever-born children (Log-rank test: 8.28, p-value = 0.02). Regarding the preceding birth interval in months, only 3602 (36.76%) of mothers had more than 35 months of spacing (Log-rank test: 70.36, p-value = 0.001) (Table 1).

**Table 1:** Household and parental characteristics of under-five children in rural Ethiopia based on the 2016 EDHS.

Household and parental characteristics	Frequency	Percentage	Chi-square for Log-rank test	P-value
**Age of mother in years**				
15–19	359	3.67	6.64	0.3551
20–24	1875	19.11		
25–29	2874	29.31		
30–34	2203	22.46		
35–39	1587	16.19		
40–44	672	6.85		
45–49	237	2.41		
**Age at first sex in years**				
<18	6726	68.58	0.04	0.8456
> = 18	3081	31.42		
**Age at first birth in years**				
<20	6503	66.31	0.35	0.5536
> = 20	3304	33.69		
**Education status of mothers**				
Not educated	6958	70.95	5.15	0.1614
Primary	2561	26.12		
Secondary	250	2.55		
College and above	38	0.38		
**Occupation status of mothers**				
Working	4178	42.60	0.61	0.4353
Not working	5629	57.40		
**Marital status**				
Married or living together	9348	95.32	1.89	0.1689
Not married	459	4.68		
**Total number of children ever born**				
1–2	2523	25.73	8.28	0.0159
3–5	3954	40.32		
> = 6	3330	33.95		
**Preceding birth interval in months**				
First birth	1647	16.81	70.36	0.0001
<24	1818	18.55		
24–35	2732	27.88		
>35	3602	36.76		
**History of abortion**				
Yes	870	8.88	4.80	0.0285
No	8937	91.12		
**Sex of household head**				
Male	8591	87.60	0.06	0.8068
Female	1216	12.40		
**Wealth of household**				
Poorest	2572	26.23	10.22	0.0368
Poorer	2497	25.46		
Middle	2254	22.97		
Richer	1934	19.73		
Richest	550	5.61		
**Household water source**				
Improved	4996	50.95	2.93	0.0872
Unimproved	4811	49.05		
**Latrine type**				
Improved	527	5.38	4.37	0.0365
Unimproved	9280	94.62		
**Covered by health insurance**				
No	9483	96.69	8.88	0.0029
Yes	324	3.31		
**Contraceptive use**				
Yes	2759	71.87	8.37	0.0038
No	7048	28.13		

### Characteristics of the children

The total number of under-five children included in the analysis were 9,807. From those, 5092 (51.92%) were female (Log-rank test: 14.26, P-value<0.001) and 251 (2.56%) were twins (Log-rank test: 125.59, P-value<0.001). Regarding breastfeeding status, 488 (4.97%) children were not breastfeeding at all (Log-rank test: 736.27, P-value<0.001). Only 2060 (21.00%) of children under the age of five were born in health facilities (Log-rank test: 4.63, P-value = 0.032). Concerning the size of a child at birth, 1684 (17.17%) of children were very large at birth and 4043 (41.23%) were average size at birth (Log-rank test: 18.05, P-value = 0.001) (Table 2).

**Table 2:** Characteristics of under-five children included in the analysis based on the 2016 EDHS.

Children characteristics	Frequency	Percentage	Chi-square for Log-rank test	P-value
**Age of a child in months**				
0–11	2050	20.90	1.89	0.3892
12–23	1877	19.14		
24–59	5880	59.96		
**Sex of a child**				
Male	5092	51.92	14.26	0.0002
Female	4715	48.08		
**Size of a child at birth**				
Very large	1684	17.17	18.05	0.0012
Larger than average	1380	14.07		
Average	4043	41.23		
Smaller than average	1016	10.36		
Very small	1684	17.17		
**Type of pregnancy**				
Single	9556	97.44	125.59	0.0001
Multiple	251	2.56		
**Place of birth**				
Home	7747	79.00	4.63	0.0315
Health facility	2060	21.00		
**Birth order**				
First	1647	16.80	3.84	0.2796
2–3	2865	29.21		
4–5	2413	24.60		
> = 6	2882	29.39		
**Breastfeeding**				
Yes	9319	95.03	736.27	0.0001
No	488	4.97		

### Time to under-five mortality (Kaplan-Meier estimates of failure function)

**Fig 2:**
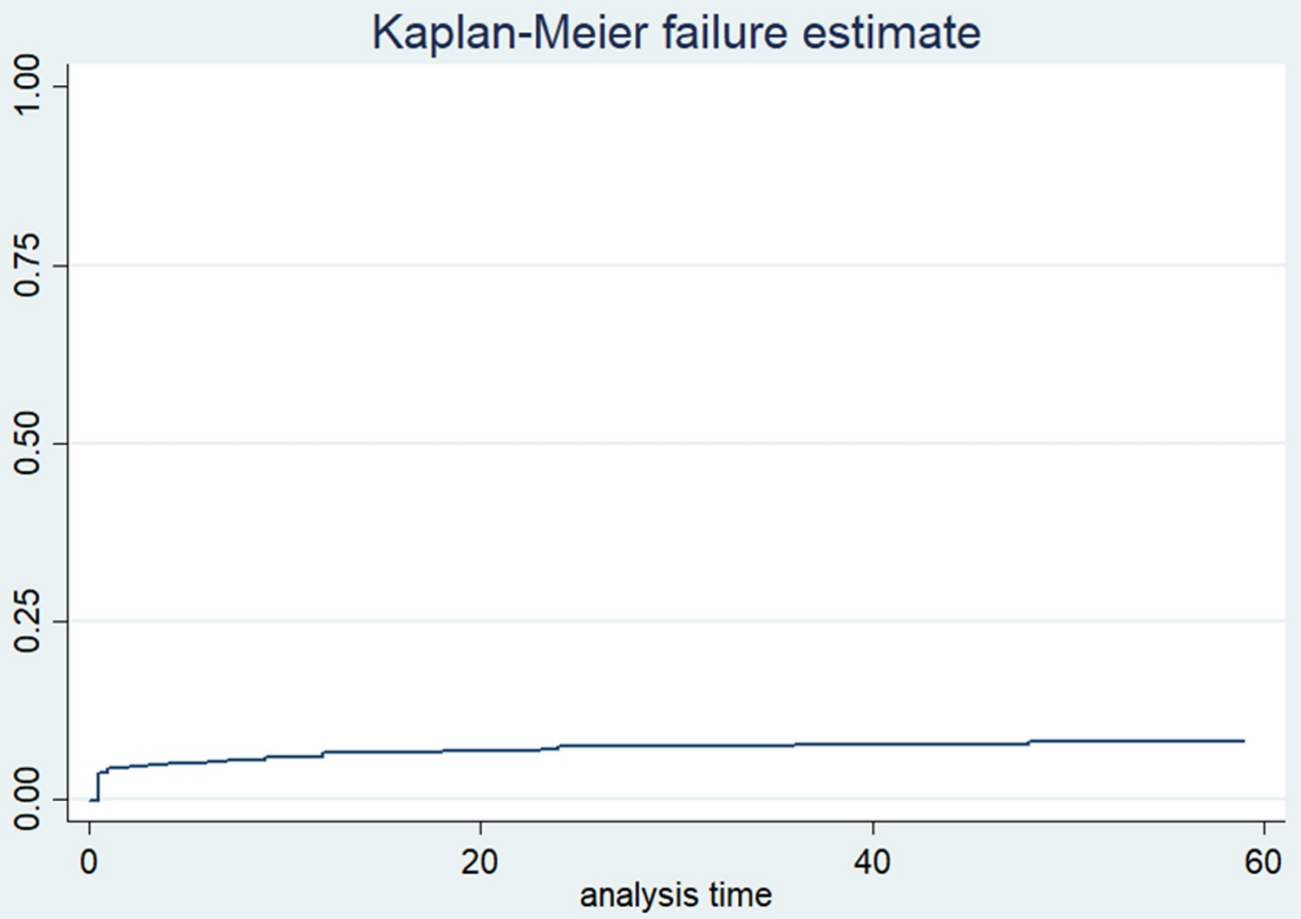
Kaplan-Meier estimates of failure function of under-five mortality in rural Ethiopia.

In this study, the participants were assessed for 272,291 person-months retrospectively. The analysis indicated that 3.31% (95% CI: 2.97, 3.68) and 5.13% (95% CI: 4.70, 5.60) of children died before 28 days and before celebrating their first birthday, respectively, in rural Ethiopia (Table 3). Similarly, as it is illustrated in the figure below, the result of the Kaplan-Meier estimate showed that 6.69% of children died before celebrating their fifth birthday in rural Ethiopia (95% CI: 6.13, 7.30) (Fig 2).

**Table 3:** A list of Kaplan-Meier estimates of the failure function (time to under-five mortality) in rural Ethiopian children under the age of five.

Time in months	Beginning total	Numbers of fail	Failure function	Time in months	Beginning total	Numbers of fail	Failure function
0	9807	273	0.0278	30	4615	0	0.0594
1	9333	51	0.0331	31	4481	0	0.0594
2	9125	24	0.0357	32	4316	0	0.0594
3	8933	7	0.0365	33	4151	0	0.0594
4	8754	16	0.0383	34	4031	0	0.0594
5	8555	10	0.0394	35	3903	0	0.0594
6	8368	15	0.0411	36	3778	16	0.0635
7	8178	9	0.0421	37	3589	0	0.0635
8	7996	13	0.0436	38	3411	0	0.0635
9	7831	14	0.0454	39	3239	0	0.0635
10	7672	6	0.0462	40	3057	0	0.0635
11	7541	5	0.0468	41	2890	0	0.0635
12	7378	35	0.0513	42	2754	0	0.0635
13	7145	6	0.0521	43	2589	0	0.0635
14	6951	0	0.0521	44	2472	0	0.0635
15	6783	2	0.0524	45	2359	0	0.0635
16	6614	2	0.0527	46	2233	0	0.0635
17	6462	0	0.0527	47	2084	0	0.0635
18	6345	8	0.0539	48	1961	7	0.0669
19	6204	3	0.0543	49	1766	0	0.0669
20	6044	0	0.0543	50	1540	0	0.0669
21	5901	0	0.0543	51	1356	0	0.0669
22	5785	0	0.0543	52	1177	0	0.0669
23	5674	5	0.0552	53	1034	0	0.0669
24	5545	25	0.0594	54	862	0	0.0669
25	5333	0	0.0594	55	703	0	0.0669
26	5178	0	0.0594	56	555	0	0.0669
27	5020	0	0.0594	57	441	0	0.0669
28	4867	0	0.0594	58	297	0	0.0669
29	4748	0	0.0594	59	147	0	0.0669

The incidence of under-five mortality was 2.70 per 100 (95% CI: 2.35, 3.12) person-months; of all deaths, the median time to death was 27 months (IQR: 12, 43 months).

### Predictors of under-five mortality in rural Ethiopia

After keeping the effect of cluster and other confounding variables, the Cox-gamma shared frailty model indicated that sex of a child, total number of children ever born, children’s size at birth, type of pregnancy, breastfeeding, type of latrine, and health insurance coverage were significant independent predictors of under-five mortality.

Keeping the effect of cluster and other variables constant, the mortality rate of female children was reduced by 38% before celebrating their fifth birthday as compared to males (AHR = 0.62, 95% CI: 0.52, 0.75). Similarly, those children who resided in households covered by health insurance were 71% at a lower risk of death than their counterparts (AHR = 0.29, 95% CI: 0.12, 0.70).

Keeping the effect of cluster and other variables constant, the mortality hazard of children living in a household with an unimproved latrine was increased by 3.44 times when compared to having an improved latrine (AHR = 3.44, 95% CI: 1.91, 6.17).

Again, the hazard of child mortality for children who had never been breastfed and had a twin pregnancy was increased by 11 times (95% CI: 9.03, 14.12) and 3.5 times (95% CI: 2.47, 4.88) as compared to children who had been breastfed and had no twin pregnancy, respectively.

Keeping the effect of cluster similar and other variables constant, those children who had extreme sized at birth (very large or very small) also had a greater hazard of death than average size babies. Those children who had very large and very small sizes at birth were 1.33 (95% CI: 1.03, 1.71) and 1.41 (95% CI: 1.10, 1.82) times more likely to die than average.

Lastly, the risk of under-five mortality was 1.4 times higher among children whose mothers had born more than five children (AHR = 1.40, 95% CI: 1.07, 1.83) (Table 4).

**Table 4:** Cox-gamma shared frailty model for predictors of under-five mortality in rural Ethiopia, EDHS 2016.

Variables	Model 0	CHR (95% CI)	Model 1 AHR (95% CI)	Model 2 AHR (95% CI)	Model 3 AHR (95% CI)
**Individual-level variables**					
**Educational status of a mother**					
College and above		1			1
Secondary		0.73 (0.21, 2.56)	0.63 (0.15, 2.60)		1.15 (0.26, 5.05)
Primary		0.44 (0.14, 1.43)	0.59 (0.14, 2.44)		0.56 (0.14, 2.28)
No education		0.53 (0.16, 1.68)	1.52 (0.36, 6.36)		0.58 (0.14, 2.35)
**Household wealth**					
Poorest		1			1
Poorer		0.94 (0.72, 1.21)	1.06 (0.81, 1.38)		1.12 (0.85, 1.50)
Middle		0.92 (0.71, 1.21)	0.98 (0.74, 1.30)		1.06 (0.78, 1.43)
Richer		1.26 (0.97, 1.65)	1.13 (0.85, 1.51)		1.23 (0.89, 1.70)
Richest		0.75 (0.48, 1.78)	0.78 (0.48, 1.26)		0.87 (0.53, 1.44)
**Type of latrine**					
Improved		1			1
Not improved		2.45 (1.40, 4.27)	3.30 (1.85, 5.88)		3.44 (1.91, 6.17)*
**Covered by health insurance**					
No		1			1
Yes		0.29 (0.12, 0.98)	0.30 (0.13, 0.73)		0.29 (0.12, 0.70)*
**Total number of children ever born**					
<3		1			
3–5		1.14 (0.90, 1.45)	1.16 (0.90, 1.50)		1.18 (0.91, 1.52)
>5		1.53 (1.21, 1.93)	1.39 (1.06, 1.81)		1.40 (1.07, 1.83)*
**Age of child in months**					
24–59		1			**1**
12–23		1.01 (0.81, 1.27)	0.94 (0.74, 1.19)		0.94 (0.74, 1.19)
0–11		0.77 (0.59, 1.01)	0.90 (0.68, 1.20)		0.89 (0.67, 1.19)
**Sex of a child**					
Male		1			1
Female		0.61 (0.51, 0.73)	0.62 (0.52, 0.74)		0.62 (0.52, 0.75)*
**Size of a child at birth**					
Average		1			1
Very large		1.23 (0.96, 1.56)	1.32 (1.03, 1.70)		1.33 (1.03 1.71)*
Larger than average		1.01 (0.77, 1.32)	1.00 (0.76, 1.32)		1.00 (0.76, 1.33)
Smaller than average		1.17 (0.87, 1.58)	1.25 (0.92, 1.69)		1.24 (0.92, 1.68)
Very small		1.41 (1.11, 1.78)	1.45 (1.13, 1.85)		1.41 (1.10, 1.82)*
**Type of pregnancy**					
Single		1			1
Multiple		5.80 (4.30, .82)	3.45 (2.45, 4.86)		3.48 (2.47, 4.88)*
**Breastfeeding**					
Yes		1			
No		11.90 (9.80, 14.70)	11.08 (8.96, 13.71)		11.29 (9.03, 14.12)*
**Community-level variables**					
**Proportion of unemployed women**					
Low		1			1
High		1.03 (0.79, 133)		0.96 (0.73, 1.27)	0.95 (0.71, 1.27)
**Proportion of not breastfeed children**					
Low		1			
High		1.71 (1.32, 2.21)		1.74 (1.33, 2.28)	0.96 (0.71, 1.30)
**Proportion of households accessed for improved water**					
Low		1			1
High		1.00 (0.77, 1.30)		1.05 (0.79, 1.39)	1.05 (0.78, 1.41)
**Proportion of women educated secondary and above**					
Low		1			1
High		0.86 (0.64, 1.15)		0.88 (0.65, 1.20)	0.81 (0.58, 1.13)
**Proportion of teenage pregnancy**					
Low		1			1
High		0.95 (0.73, 1.22)		0.97 (0.75, 1.27)	0.93 (0.71, 1.24)
**Proportion of households with an unimproved latrine**					
Low		1			1
High		0.98 (0.74, 1.29)		0.96 (0.72, 1.30)	0.85 (0.62, 1.17)
**Proportion of households above middle wealth**					
Low		1			1
High		0.89 (0.69, 1.16)		0.96 (0.71, 1.30)	0.97 (0.70, 1.35)
**Proportion of women not using contraception**					
Low		1			1
High		1.25 (0.94, 1.65)		1.20 (0.84, 1.70)	1.18 (0.82, 1.71)
**Region**					
Tigray		1			1
Afar		1.79 (0.77, 4.16)		1.49 (0.60, 3.75)	1.29 (0.50, 3.32)
Amhara		1.04 (0.62, 1.74)		1.12 (0.65, 1.93)	1.11 (0.63, 1.97)
Oromia		1.13 (0.70, 1.84)		1.01 (0.60, 1.70)	0.89 (0.51, 1.53)
Somali		1.40 (0.76, 2.58)		0.90 (0.44, 1.86)	0.95 (0.45, 2.03)
Benishangul		1.46 (0.62, 3.42)		1.21 (0.51, 2.87)	1.01 (0.42, 2.47)
SNNPs		1.11 (0.67, 1.84)		1.00 (0.59, 1.69)	0.76 (0.44, 1.33)
Gambela		1.63 (0.27, 9.66)		1.37 (0.23, 8.27)	1.07 (0.17, 6.60)
Harari		1.74 (0.31, 9.79)		1.54 (0.27, 8.79)	1.30 (0.22, 7.52)
Dire Dawa		1.76 (0.39, 7.80)		1.47 (0.32, 6.75)	1.43 (0.30, 6.77)

CHR = Crude Hazard Ratio, SNNPs = Southern Nation Nationalities and Peoples, 1 = reference and * = statistically significant (P-value less than 0.05).

#### Model fitness and adequacy test

**Fig 3:**
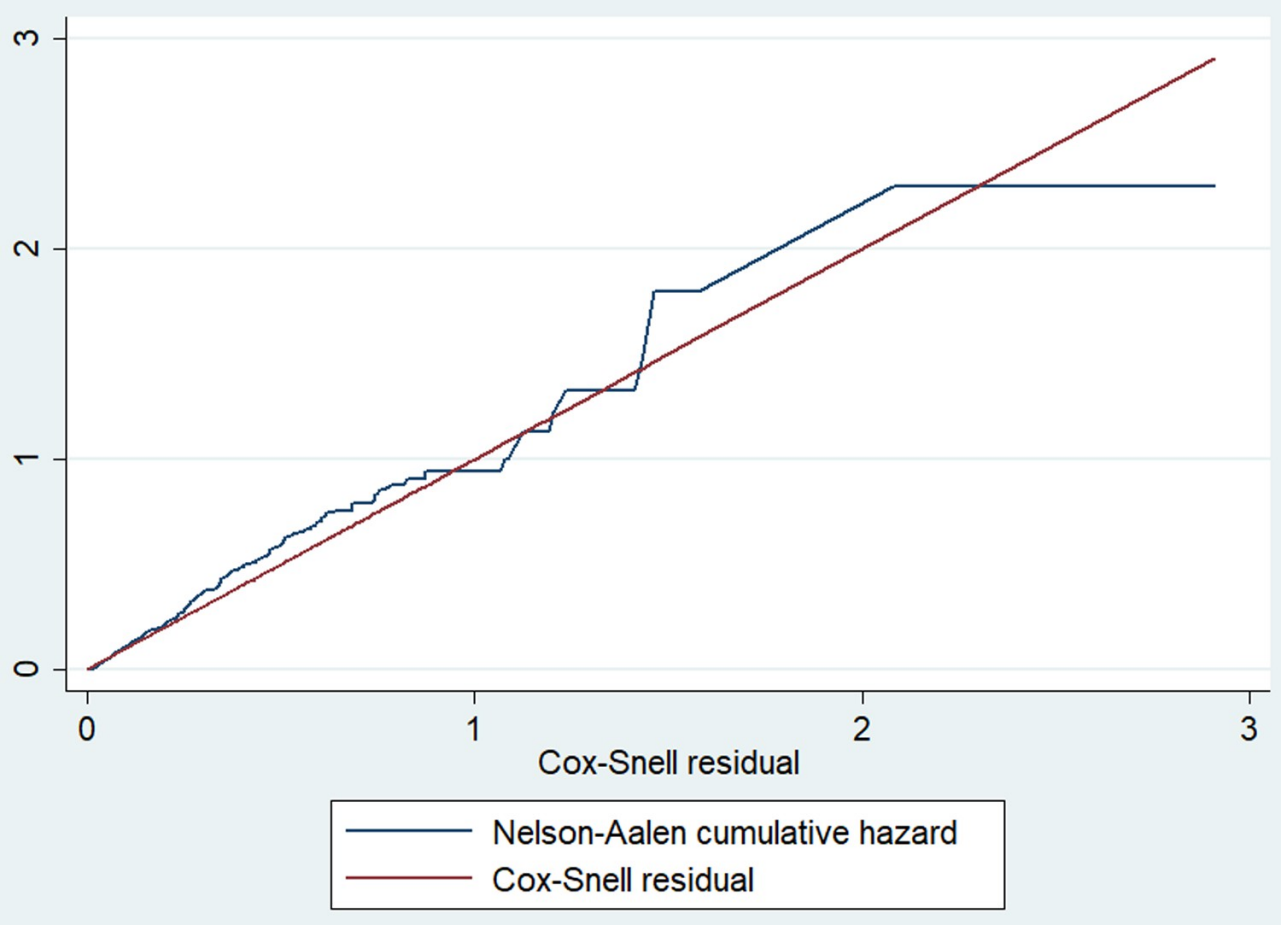
Cox-Snell residuals plot for fitting Cox-model for both individual and community-level predictors of under-five mortality in rural Ethiopia.

The Cox-Snell residuals plot for fitting the Cox model indicated that the final model was the best fit with the data than the other preceding models (Fig 3) and (Table 5).

**Table 5:** Model fitness and adequacy test statistics for all level predictors of under-five mortality in rural Ethiopia.

Model fitness and adequacy test	Null model	Model 1	Model 2	Model 3
Log-likelihood	-4941.03	-4659.07	-4930.16	-4654.72
LR test for deviance	1	0.00001	0.00001	0.00001

1 = Reference.

## Discussion

The analysis indicated that 3.31%, 5.13%, and 6.69% of children died before 28 days, before celebrating their first and fifth birthdays, respectively, in rural Ethiopia. After keeping the effect of cluster and other confounders, the result indicated that sex of a child, the total number of children ever born, children size at birth, type of pregnancy, breastfeeding, type of toilet, and health insurance coverage were significant predictors of under-five mortality.

The finding of neonatal mortality was similar to a study conducted in Jimma, Ethiopia (3.55%) [36]. It is also similar to a study conducted in Ethiopia (3.30%) [37]. The finding was lower than a study conducted in Gondar (14.30%) [38], Tigray (6.30%) [39], and Somali (5.70%) [40]. It is also lower than a study conducted in Nigeria (4.10%) [41] and Pakistan (4.73%) [42]. The discrepancy could be caused by time differences. Moreover, it may be due to the difference in sociocultural contexts, especially concerning the economic status of the population and cultural mal-practices during and after delivery as well as postpartum.

The percentage of infant mortality was similar to a study conducted in rural Ethiopia (4.70%) [43]. But it is lower than a study conducted in Southwest Ethiopia (9.70%) [44] and Ethiopia (11.00%) [45]. It is also lower than a study conducted in Amhara, Ethiopia (8.80%) [46] and Ethiopia (5.90%) [47]. Again, this is lower than a study conducted in Sidama Zone (9.6.%) [48]. But it was higher as compared to a study conducted in Nepal (3.21%) [49]. This difference may be due to the fact that as time goes different advancements in disease management as well as increased access to different health facilities in the current study and not in previous studies of Ethiopia. The finding of under-five mortality was in line with studies conducted in Ethiopia (6.70% to 6.72%) [27, 50]. The finding was also similar to a study conducted in Tanzania (7.04%) [51]. But, the rate of under-five mortality was higher as compared to a study conducted in Ethiopia (6.00%) [12]. The finding was also higher than a study conducted in Ghana (4.91%) [15] and Bhutan (3.70%) [52]. The possible reason for this discrepancy might be differences in socio-cultural and other contextual factors.

Whereas it was lower than studies conducted in Ethiopia (7.44% to 10.00%) [11, 53]. The finding was also lower than a study conducted in South Sudan (10.10%) [54] and Bengal (9.69%) [17]. The discrepancy might be due to time variation as well as differences in infrastructure, and the health service strategies may not be similar with Bengal.

Those female children were 38% less likely to die before celebrating their fifth birthday as compared to males. The finding was in agreement with studies conducted in Ethiopia [11, 12, 16, 20, 27, 50, 55, 56] and our non-parametric Log-rank test also revealed a significant difference between females and males (Table 2). The result was also similar to studies conducted in Malawi [57], Ghana [15], and Nigeria [14, 58]. It is also supported by a study conducted in Sierra Leone [13] and Tanzania [51]. The possible reason for this association may be biological differences like hormones circulating in the bloodstream and natural protection mechanisms for different diseases. So, it implies that male new borne need special attention in the adaptation of the new environment, and their sickness required vigorous management due to its deadly outcome.

Similarly, the risk of death in children who resided in a household covered by health insurance was 71% less than their counterparts. This was supported by our descriptive result that the Log-Rank test showed a significant difference in under-five mortality between those households covered by health insurance and those not covered (Table 1). This may be due to the fact that most rural people may fear the cost of health care and may have poor health-seeking behavior for childhood illness. This implies the requirement of intensive efforts to enhance health insurance coverage in the country to reduce child mortality.

The hazard ratio of children who lived in a household with an unimproved latrine was increased by 3.44 fold as compared to the improved. The finding was in agreement with a study conducted in Bengal [17]. It was also supported in a study conducted in Bhutan [52] and Nepal [59]. The association could be due to the fact that unimproved latrines were a source of infections such as intestinal parasites and other diarrheal diseases. Diarrheal diseases are the third leading cause of child mortality in the country [60]. So, enhancing improved latrine and its utilization must be one focus of child mortality programs in the country.

The hazard of children mortality that had never been breastfed was increased by 11 times more than their counterparts. The finding was supported by a study conducted in Ethiopia [16, 19, 29]. It was also in agreement with a study conducted in Indonesia [61]. This might be due to mal-absorption of foods in the first 6 months, which may lead them to infection. Moreover, they lack natural antibodies (i.e., antibodies passed through breast milk) for infection prevention. This implies that in the countries like Ethiopia where infectious diseases are the leading cause of child mortality [60], breastfeeding is an option-less intervention in child mortality. The hazard ratio (risk of death) of multiple pregnancies was increased by 3.5 times as compared to a single. The finding was in agreement with studies conducted in Ethiopia [12, 18, 19, 27, 29, 55, 56]. It was also supported by a study conducted in Ghana [15]. It was again supported by a study conducted in Rwanda [62]. It is obvious that most multiple births are premature and which is the second cause of child mortality next to sepsis [60]. The possible reason for this association may be that different medical assistance during labour and postpartum may increase the risk of death as most of the Ethiopian health facilities are not fully equipped with all the necessary materials [63]. Moreover, it may be due to differences in physiological and pathological processes like twin-twin transfusion syndrome. So that mothers and health professionals need to give particular attention to multiple births to prevent child mortality of morbidity.

Children born in extreme sizes (very large and very small) had a higher risk of dying than the general population. The result was supported by a study conducted in Ethiopia [18, 27, 29, 50]. The result of this study was congruent with studies conducted in Malawi [57], Tanzania [64], and Nigeria [14, 58]. It is also supported by a study conducted in Sierra Leone [13]. This might be due to the fact that very small births were exposed to frequent infections. In contrast, very large babies were subjected to different birth traumas during delivery.

The risk of under-five mortality was higher among children whose mothers had born more than five children. The result was in agreement with a study conducted in Sierra Leone [13]. The finding was also supported by a study conducted in Nigeria [14]. The possible reasons may be that mothers may have less attention to children and the children may not get enough food and safe health care. Therefore, policymakers better focus on the prevention of grand parity through determining the number of children per woman to reduce child mortality.

Despite the Ethiopian government have been working to reduce neonatal, infant, and under-five mortality as a primary concern, in reality, significant numbers of neonates, infants, and under-five children died before 28 days, before celebrating their first and fifth birthdays, respectively, in rural Ethiopia. So, different concerned bodies (policymakers, planners, health care providers) need to be focused on the above mentioned statistical factors (sex of a child, total number of children ever born, children’s size at birth, type of pregnancy, breastfeeding, type of toilet, health insurance coverage) to achieve Sustainable Developmental Goals.

## Strength and limitation of the study

The study has the following strengths: utilizes nationwide data that can easily detect small differences (effect size), considering the effect of clustering (the data recorded in the same cluster were correlated), and time to under-five mortality is time to the event of interest. Therefore, the model (cox-gamma shared frailty model) accounts for both the hierarchical nature of the data and time to the event of interest including censoring. However, it is not without limitations. The cross-sectional nature of the study restricts to build a causal and effect relationship. Moreover, as the analysis was based on secondary data, certain variables that may be potential factors for under-five mortality were missed.

## Conclusion

Still now, neonatal, infant, and under-five mortality were very high in rural Ethiopia. In the final adjusted model, being ever born from a mother who had greater than five children, very small or very large children’s size at birth, twin pregnancy, not having improved latrine, and not breastfeeding were positively associated with under-five mortality. However, being female sex, and covered by health insurance were negatively associated with under-five mortality. Further emphasis should be given to twin and non-breastfeeding children as well as households better to encourage membership of community health insurance and utilization of improved latrines.

## List of abbreviations

AHR: Adjusted Hazard Ratio
CHR: Crude Hazard Ratio
CSA: Central Statistical Agency
EDHS: Ethiopia Demographic and Health Survey
U5M: Under-five Mortality
